# Human placental methylome in the interplay of adverse placental health, environmental exposure, and pregnancy outcome

**DOI:** 10.1371/journal.pgen.1008236

**Published:** 2019-08-01

**Authors:** Amanda Vlahos, Toby Mansell, Richard Saffery, Boris Novakovic

**Affiliations:** 1 Epigenetics Group, Murdoch Children’s Research Institute, Royal Children’s Hospital, Parkville, Victoria, Australia; 2 Department of Paediatrics, University of Melbourne, Parkville, Victoria, Australia; University of Pennsylvania, UNITED STATES

## Abstract

The placenta is the interface between maternal and fetal circulations, integrating maternal and fetal signals to selectively regulate nutrient, gas, and waste exchange, as well as secrete hormones. In turn, the placenta helps create the in utero environment and control fetal growth and development. The unique epigenetic profile of the human placenta likely reflects its early developmental separation from the fetus proper and its role in mediating maternal–fetal exchange that leaves it open to a range of exogenous exposures in the maternal circulation. In this review, we cover recent advances in DNA methylation in the context of placental function and development, as well as the interaction between the pregnancy and the environment.

## Introduction

Establishing a successful pregnancy depends on a complex sequence of interactions between maternal and fetal cells, and their interlocutory signals [1]. This interaction takes place on many levels, from systematic endocrine factors to direct paracrine and juxtacrine contact between the placenta and maternal decidual cells. The placenta is a major transient endocrine organ that plays a key role in enabling many of these complex interactions. Placental trophoblasts extensively remodel the maternal decidua and uterine vasculature to ensure appropriate blood flow for the exchange of nutrients and waste between fetal and maternal circulations. In turn, this creates a unique environment at the fetal–maternal interface [2].

The extent to which the placenta invades the maternal decidua and interacts with the maternal circulation depends on the structure of the placenta. Evolutionarily, the placenta is one of the most diverse organs, evolving independently in multiple lineages and with different placental patterning [3]. The hemochorial human placenta is unique from other primates and model organisms like mice in that trophoblast invasion is more extensive and implantation is interstitial [4]. The placenta differentiates from the extraembryonic trophectoderm, giving rise to villous cytotrophoblasts (VCTs) that can proliferate or fuse to form the multinuclear syncytiotrophoblast (ST) layer, which acts as the barrier between maternal and fetal circulations, and the extra-villous trophoblasts (EVTs), which invade the decidua and remodel maternal spiral arteries [5]. Placental cell composition and function are dynamic over gestation, with a major change being the increased flow of maternal blood into the intravillous space around the transition from first to second trimester, which results in a change in oxygen concentration and extensive remodelling of villous structure [6, 7]. This interaction of maternal and fetal cells is facilitated by considerable adaptation of the maternal immune response, ensuring tolerance of the developing fetus while retaining capability for host cell defence and contributing to healthy development [8].

Compelling evidence from human epidemiological studies suggests that adverse exposures in utero can alter the intrauterine environment, with potential implications for fetal development and long-term offspring health [9]. Due to its role as interface between maternal and fetal circulations, the placenta is constantly exposed to exogenous factors that are present in the maternal circulation, with the potential for these exposures to leave a lasting molecular ‘footprint’ on the placenta. In keeping with this, it is not surprising that the extraembryonic origin, relatively short life span, and ongoing direct exposure to all exogenous signals are reflected in a unique epigenetic profile [10]. Here, we highlight and discuss emerging knowledge on the unique features of the human placental methylome (total DNA methylation profile) and summarise research linking in utero exposures, adverse placental health, and fetal outcomes to altered DNA methylation in the human placenta. We go on to discuss the potential impact of single-cell techniques on understanding the placental methylome and how this will pave the way for future use of DNA methylation in the placenta as a biomarker to diagnose placental syndromes and predict fetal outcomes.

### DNA methylation remodelling in extraembryonic cells

Epigenetics refers to the molecular interactions that influence DNA packaging into chromatin, thereby regulating DNA accessibility and gene expression. Epigenetic modifications are added either directly to DNA (as in DNA methylation) or to the amino acid tails of histones that constitute the nucleosome around which DNA is coiled.

**Fig 1:**
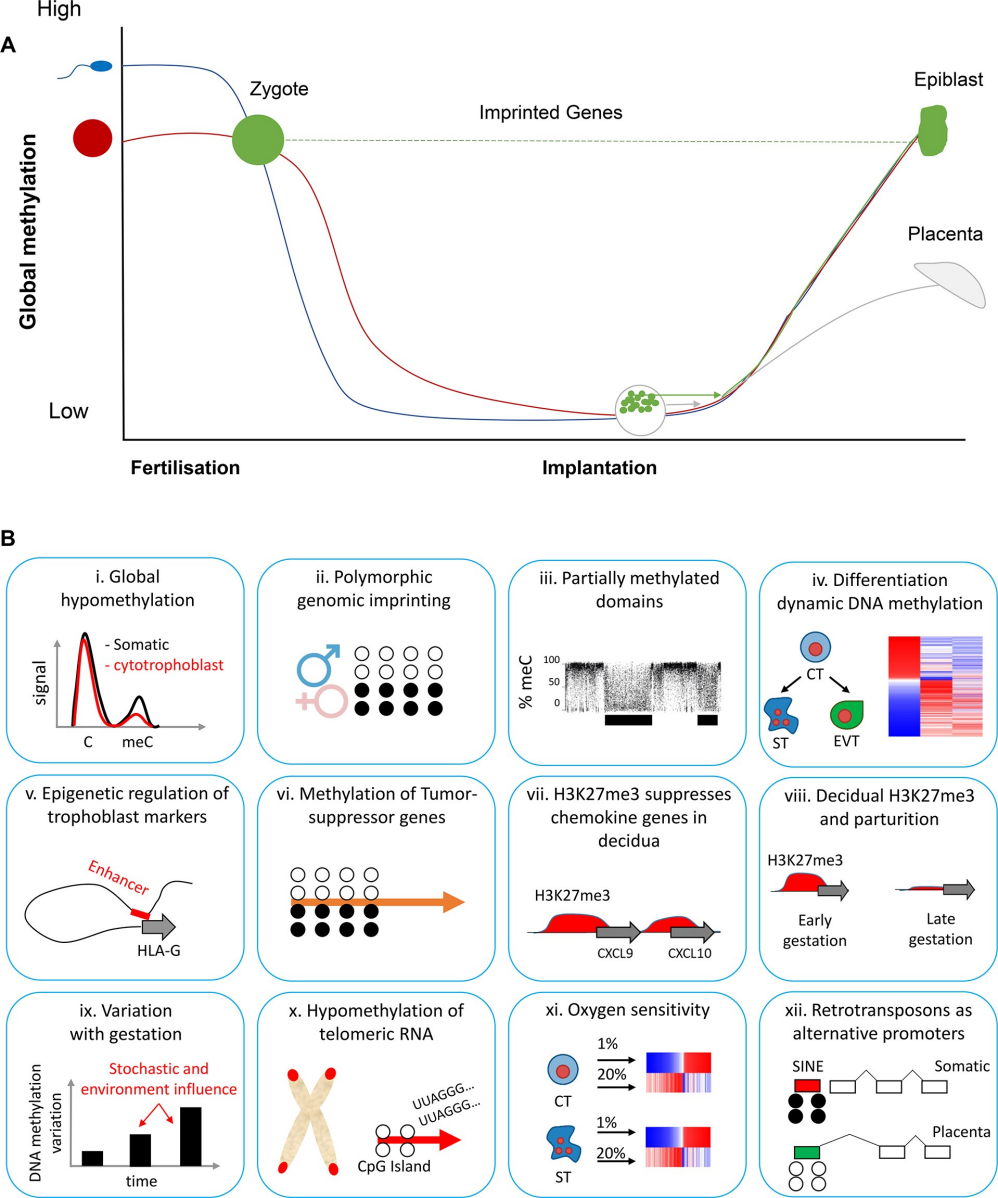
Unique epigenetic features of the human placenta. **(A)** DNA methylation dynamics during early human development. After fertilisation, the sperm and egg undergo a wave of hypomethylation, except at imprinted genes. Around implantation, the inner cell mass of the blastocyst, which develops into the epiblast, and the trophoblast, which develops into the placenta, undergo de novo methylation to differing extents. **(B)** Unique features of the placenta methylome arising as a result of the remethylation differences in **A**. B (i) Global hypomethylation [15, 16], (ii) polymorphic imprinting [17], (iii) PMDs [18, 19], (iv) trophoblast differentiation dynamics [13], (v) epigenetic regulation of HLA-G [20], (vi) tumour suppressor promoter methylation [21, 22], (vii) silencing of chemokines by a repressive histone mark, H3K27me3 [23], (viii) control of parturition by H3K27me3 in decidua [23], (ix) interindividual variation increases with gestation [24], (x) TERRA promoter hypomethylation [25], (xi) trophoblast DNA methylation is sensitive to oxygen concentration [14], (xii) hypomethylated retrotransposons used as alternative promoters [26, 27]. C, cytosine nucleotide; CpG, cytoside-guanine dinucleotide; CT, cytotrophoblast; CXCL9, C-X-C motif chemokine ligand 9; CXCL10, C-X-C motif chemokine ligand 9; EVT, extra-villous trophoblast; HLA-G, major histocompatibility complex, class I, G; H3K27me3, histone 3 Lysine 27 trimethylation; meC, methylated cytosine; PMD, partially methylated domain; SINE, short interspersed nuclear element; ST, syncytiotrophoblast; TERRA, telomere RNA.

DNA methylation is the most widely studied and well-characterised epigenetic modification. It involves the addition of a methyl group to a cytoside within a cytoside-guanine dinucleotide (CpG site) [11]. CpG sites often cluster together in CpG islands close to the promoter and regulatory regions of genes, and within this context increased DNA methylation blocks access to the underlying DNA sequence, leading to reduced gene expression. During human preimplantation development, DNA methylation is highly dynamic (Fig 1A). Notably, at the blastocyst stage, the inner cell mass and trophectoderm undergo a wave of de novo DNA methylation, but to different extents, leading to differences in final global DNA methylation levels [12].

DNA methylation continues to be dynamic in trophoblast cells after implantation and throughout gestation, with the process of VCTs’ differentiation to ST and the acquisition of an invasive phenotype in EVT, both of which involving widespread DNA methylation changes [13]. Interestingly, culture in a low oxygen concentration induced opposing DNA methylation change at certain differentiation-associated differentially methylated regions (DMRs) and was associated with lower differentiation potential, an effect potentially modulated by the activator protein 1 (AP-1) transcription factor complex [14]. These findings highlight the intimate relationship between DNA methylation patterns, trophoblast differentiation, and the sensing of environmental signals.

### Placental global hypomethylation is not specific to repetitive elements

The aforementioned differences in DNA methylation re-establishment result in the placenta exhibiting a unique, globally hypomethylated DNA methylation profile compared with normal somatic tissue (Fig 1B) [15]. High-performance liquid chromatography (HPLC) analysis of the total 5-methylcytosine content estimates that human placental tissue is on average 2.5%–3% methylated compared to approximately 4% in human cord blood (Fig 1B(i)). Repetitive elements, which cover approximately 35% of the human genome, are supressed with elevated levels of DNA methylation [28, 29]. Earlier studies of the placental methylome found that some repetitive elements, such as long interspersed element-1 (*LINE-1*) [30, 31]) and human endogenous reterovirus (*HERV*) [32], are key DMRs in placental tissue. Placental hypomethylation of these repetitive elements is thought to regulate placenta-specific functions. For example, some retrotransposons function as alternative promoters for placental-specific transcripts, such as potassium voltage-gated channel subfamily H member 5 (*KCNH5*) and interleukin 2 receptor, beta 1 subunit (*IL2RB*) (Fig 1B(xii)) [26, 27, 32]. Expression of an alternative form of *KCNH5* may contribute to trophoblast invasiveness, a conclusion that is supported by the finding that the placenta-specific transcript and associated hypomethylated promoter occur frequently in melanoma cells, but never in healthy somatic cell types [33]. IL2RB is a subunit of the IL2 receptor and is expressed primarily by lymphocytes, and the function of the trophoblast specific-transcript is currently unknown [27]. In fact, despite the widespread potential for retrotransposon-driven novel transcription in the placenta, relatively little is known about its potential role in correct placental functioning.

Recent findings have dispelled the notion that retrotransposons are specifically hypomethylated in the placenta. In a landmark study, Schroeder and colleagues [18] showed that placental DNA hypomethylation mainly occurs within partially methylated domains (PMDs)—that is, large stretches (up to 100 kb) of gene-poor regions with reduced DNA methylation that are interspersed with regions of higher DNA methylation (Fig 1B(iii)). Using Methyl-C sequencing, it was estimated that PMDs covered approximately 37% of the placental genome and that genes within placental PMDs have placental-specific functions. An important observation was that *LINE-1* elements located in a PMD showed the same level of methylation as the surrounding region (approximately 40%), while *LINE-1* elements outside PMDs were not hypomethylated (approximately 80% methylation). Likewise, another study used reduced representation bisulfide sequencing (RRBS) to show that placental hypomethylation is enriched at CpG-poor intergenic regions and gene bodies, and that retrotransposons in these regions were, in fact, slightly more methylated than non-retrotransposon elements [34]. Taken together, this suggests that while the placenta may utilise unmethylated retrotransposon elements as alternative promoters, these regions do not solely explain the function global hypomethylation in the placenta. Further research into PMDs may reveal new insights into their function, although role of PMDs in placenta is yet to be determined.

### Placental pseudomalignancy: An epigenetically regulated developmental mechanism co-opted by cancers?

The similarities between placentation and cancer were first pointed out in 1902 [35]. In the 1970s and 1980s, studies showed that the human placenta expressed high levels of oncogenes [36], while cancers expressed high levels of placental hormones, like human chorionic gonadotropin (hCG). Thus, this led to the hypothesis that tumorogenesis was a ‘pathological recapitulation’ of normal placental development [37]. Unlike cancers, placental trophoblasts restrict their proliferative and invasive phenotype, and have therefore been described as pseudomalignant. Trophoblasts activate similar molecular and epigenetic pathways observed in cancers [38], and it has been suggested that cancers co-opt placental-specific epigenetic programming [39].

In addition to the global hypomethylation and presence of PMDs, earlier studies showed that promoters of common tumour suppressor genes are monoallelically methylated in the placenta and trophoblasts (Fig 1B(vi)) [21, 22]. These promoters become completely methylated in choricarcinomas (trophoblast cancers), supporting the notion that these cancer-like programs are carefully regulated in the placenta. Similarly, cancer-like global DNA hypomethylation blocks in early gestation placenta are lost in later gestation, suggesting a necessary switch that limits placenta invasion and other tumour-like properties in later pregnancy [40].

Studying the establishment of these pseudomalignant methylomes during early implantation is difficult in humans. As such, we do not know how a completely hypomethylated human trophectoderm develops these features. An interesting mouse study that addressed this question comes from the Meissner lab, which showed that partially methylated promoter regions are established rapidly [19]. In embryonic stem cells, these regions are marked by a repressive histone modification, histone 3 lysine 27 trimethylation (H3K27me3), indicating that polycomb group of proteins are keeping these regions closed and thereby potentially allow DNA methyltransferases (DNMTs) to methylate DNA. An important question remains: are PMDs and tumour suppressor promoter methylation drivers of placental function or bystanders?

### Novel imprinted regions in the placenta

During development, certain regions of DNA, called germline differentially methylated regions (gDMRs), maintain gamete DNA methylation during genome-wide reprogramming and throughout development [41]. These gDMRs can regulate expression at nearby genes, resulting in monoallelic parent-of-origin expression known as genomic imprinting [42]. There are multiple theories as to why genes evolved to be imprinted [43], but the prevailing hypothesis is that of the kinship theory [44]. In brief, imprinting allows for natural selection to maximise the fitness of the allele through maximising not just the health of the individual inheriting the allele but also the related individuals who will also inherit that allele from the same parent [42]. An example is a gene that regulates growth in utero, and consequently influences the fetal demand for maternal resources [45]. As multiple offspring from the same mother may have different fathers, maximising the kinship fitness bestowed by the maternally inherited allele is more likely to improve kinship fitness overall than the paternally inherited allele. As such, paternal alleles may evolve as silenced to avoid conflicts between maternal and paternal allelic expression [45]. Recent research indicates that the placenta has more gDMRs than previously thought [17, 46], which is consistent with the importance of the placenta in regulating fetal growth and maternal resource demand. However, it remains unclear whether the majority of placenta-specific gDMRs are functional in pregnancy, or whether they simply reflect the methylation status of a gamete. Hanna and colleagues [17] identified 144 gDMRs in the human placenta, in which most appeared to maintain maternal DNA methylation patterns (mDMRs). Similarly, Hamada and colleagues [46] performed combined whole-genome bisulfide sequencing and RNA-seq, which identified approximately 1,800 mDMRs in the human placenta, some of which were associated with potential paternal and maternal imprinted genes such as TP53 induced glycolysis regulatory phosphatase (*TIGAR*), sodium bicarbonate cotransporter 3 (*SLC4A7*), and zinc finger protein (*ZFP90*). The function of these imprinted genes in placenta or fetal development, however, remains to be elucidated. An interesting observation requiring follow-up study is that the DNA methyltransferase 1 (*DNMT1*) promoter itself is maternally imprinted in the human placenta [47]. As with several other aspects of human placental methylation, this promoter methylation is evolutionary conserved only in primates, not in other mammals, and does not seem to be required for global hypomethylation in the placenta [48, 49]. It is easy to speculate that maternal imprinting of *DNMT1* is a mechanism to prevent the activation of growth-promoting genes, such as insulin-like growth factor 2 (*IGF2*), by DNMT1 overexpression [50]. In line with this, *DNMT1* expression shows a modest positive correlation with placental growth [51]. However, there is no confirmed function for *DNMT1* imprinting in the placenta. A limitation of using whole placental tissue to identify novel imprinted genes is that there may be variation between different cell types, which can lead to some imprinted regions being missed. Additionally, without identifying the specific cell types that show imprinting, it is hard to study the function of these novel imprinted regions.

### In utero exposures alter the placenta methylation, which may impact placental and fetal health

DNA methylation is sensitive to environmental exposure but relatively stable once established. Consequently, studies have examined the influence of in utero exposures on DNA methylation in the placenta in the hope that these patterns could be used as potential biomarkers to diagnose placental disorders or even predict fetal outcomes. The overall level of placental tissue DNA methylation increases over gestation, as does the level of interindividual variation between placentas, potentially indicative of a response to a myriad of environmental exposures and stochastic events, or with a transition and variation in cell composition over time [24]. Many of these studies take the form of epigenome-wide association studies (EWAS), and generally use the Infinium HumanMethylation arrays [52, 53]. Mounting evidence suggests that exposures in utero influence the human placental methylome. In Tables 1 and 2, we summarise key EWAS studies using the human placenta since 2016. For a summary of studies predating 2016, please see [54]. Table 1 summarises emerging research linking in utero exposures and the human placental methylome. Given the key role of the placenta in fetal programming, altered DNA methylation profiles of the placenta may cause aberrant placental development and function, which may in turn influence fetal outcomes. Table 2 summarises emerging research linking the human placental methylome and pregnancy outcomes.

**Table 1:** Placenta epigenetics and environment.

Study	Exposure	Technique/coverage	*N*	Placenta tissue	Finding
[88]	Phthalates, cross-sectional	Infinium EPIC, EWAS	7 low exposure 5 high exposure	Placental villous tree from terminated pregnancies	39 genes differentially methylated + altered gene expression in high exposure group. *EGFR* is a key gene likely to play a key role in mediating effects on placenta.
[89]	Maternal diabetes	Infinium HM450, EWAS	17 diabetic pregnancies 17 controls	Term placenta tissue	Sex-specific DNA methylation changes in diabetic pregnancy. Total of 465 differential probes in male and 247 probes in female placenta.
[90]	Maternal lifetime stress	Infinium HM450, EWAS	207 samples with stress as a continuous variable	Term placenta tissue	112 CpGs showed significant association with maternal lifetime stress level.
[91]	Smoking at both weeks 12 and 32 of pregnancy	Infinium HM450K, EWAS	28 smokers and 151 nonsmokers in discovery cohort, 40 smokers and 208 nonsmokers in replication	Term placenta tissue	50 CpGs across 46 loci differentially methylated, 14 with difference >5%. 4 CpGs across 3 loci validated and replicated. These CpGs were also associated with urine cotinine.
[92]	Maternal glycemic response in nondiabetic pregnancies	Infinium EPIC, EWAS	448 samples with glucose response as a continuous variable with a normal distribution	Term placenta tissue	7 significant probes, 4 of which are near *PDE4B*, with methylation change below 2%. Methylation at birth was correlated with glucose levels after a 2-hour glucose test during the second trimester.
[93]	Smoking during first trimester	ELISA global methylation, pyrosequencing of *LINE-1* and *AluYb8*	23 nonsmokers, 17 smokers	Placenta tissue from terminated pregnancies	Prenatal exposure to smoking was not associated with global methylation as measured through any of the three assays. There was similarly no association in fetal liver nor small intestine tissues.
[94]	Alcohol during each trimester	MassARRAY Epityper to measure 3 CpGs in *Alu* as a marker of global methylation	187 pregnancies	Pooling of the central tissue from 3 random biopsies from placenta	A male-specific association of increased global methylation in alcohol exposure throughout all three trimesters compared with no exposure.
[95]	War trauma and chronic stress	Infinium HM450K to investigate 67 CpGs in *BDNF*, bisulphite sequencing used to validate a region	24 pregnancies	Biopsy from largest placental cotyledon	3 CpGs were associated with war stress in placenta; of those, 1 was associated with chronic stress. Associations were tissue specific, with different associations observed in maternal and cord blood.
[96]	Plastic exposure (BPA)	Restriction digestion to estimate methylation of 94 candidate genes	At least 3 independent experiments for each analysis	Cultured first trimester trophoblast cells	Generally, promoter methylation across the genes was decreased in the BPA exposed group compared with control. BPA exposure also impaired cell viability and proliferation and was associated with decreased expression of genes associated with proliferation.
[97]	Air conditions (NO_2_, PM_10_, temperature, and humidity) at multiple time points during pregnancy	Infinium HM450K, both EWAS and candidate gene approaches	668 pregnancies	Biopsy from centre of fetal side of placenta	PM_10_ was positively associated with *Alu* methylation. In EWAS analysis, NO_2_ was associated with 3 CpGs, and each of the other exposures were associated with 1 CpG. In candidate analysis, NO_2_ and PM_10_ were associated with several CpGs in a timing-specific manner, particularly in the *ADORA2B* and *PXT1* genes.
[98]	Air pollution (PM_2.5_ and PM_10_) for each trimester	High‐pressure liquid chromatography to measure global methylation	44 from low exposure region and 48 from high exposure region	Biopsy at fixed distance from umbilical cord in the fetal side of the placenta	PM_2.5_ and PM_10_ were positively associated with global methylation in the first trimester only. PM_2.5_ and PM_10_ in first trimester were also negatively associated with *SAMe* expression. Global methylation was not associated with other prenatal exposures or birth outcomes.
[99]	Cadmium	Infinium HM450K, EWAS	Cohort 1: 343 placentas Cohort 2: 141 placentas	Term placenta tissue	17 probes significantly associated with cadmium exposure in pregnancy. Inflammatory signalling and cell growth pathways affected.
[100]	GDM	LC-MS/MS, Global	56 GDM and 974 controls	Term placenta tissue	Placentas from GDM pregnancies had higher global DNA methylation compared with controls (mean 3.22% versus 3%).
[101]	Polybrominated diphenyl ethers, assessed in cord blood	Pyrosequencing, 4 CpGs in *IGF2*, 3 in *LINE-1*, and 1 in *NR3C1*	80 pregnancies	8 pooled placental biopsies	BDE-153 and BDE-209 exposure was negatively associated with *IGF2* methylation, BDE-153 was negatively associated with *NR3C1* methylation, and BDE-66 was negatively associated with *LINE-1* methylation.
[102]	Arsenic	Infinium HM450K, EWAS	343 pregnancies	Term placenta tissue	163 differentially methylated loci (FDR < 0.05), with 11 probes within the *LYRM2* gene associated with arsenic levels in placenta.
[103]	POPs	*LINE-1* as a DNA methylation global marker	109 pregnancies	Term placenta tissue	Seven POPs were measured. *LINE-1* methylation correlated with one of them: *β*-hexachlorhexane.
[104]	Proximity to major roadways	*LINE-1* and AluYb8 as global DNA methylation markers	215 pregnancies	Term placenta tissue	Proximity to major roads was associated with mean 175 g lower birth weight and a concordant lower *LINE-1* methylation, which was not causative.
[105]	Maternal prepregnancy obesity	MeDIP and Nimblegen 2.1 M Human DNA methylation array, EWAS	10 obese (mean BMI 34), 10 healthy (mean BMI 23.4)	Term placenta tissue after cesarean delivery	262 genes promoters showed an inverse relationship between DNA methylation and hydroxymethylation in association with obesity. Top pathways were pregnancy and immune response.
[106]	Particulate air pollution	Alu as a global DNA methylation marker	500 pregnancies from the ENVIRONAGE cohort	Term placenta tissue	Exposure to particulate matter was associated with increased mutation rate at Alu elements, and altered DNA methylation at promoters of tumour suppressor and DNA repair genes.
[107]	A range of self-reported exposures	WGBS, global at single base resolution	47 pregnancies	Term placenta tissue	Pesticides professionally applied outside the home associated with higher average methylation over PMDs.
[108]	Vitamin C and maternal smoking	Targeted Bisulfite Sequencing of 477 gene regions, total 79,258 CpG sites	12 controls, 6 smoking and placebo, 9 smoking and vitamin C	Term placenta tissue	DNA methylation at 458 CpG sites was altered by smoking. Vitamin C restored ‘normal’ DNA methylation at 58% of CpG sites.

Abbreviations: *ADORA2B*, adenosine A2b receptor; *AluYb8*, *Alu* Yb8-linage element; BDE, brominated diphenyl ether; *BDNF*, brain-derived neurotrophic factor; BMI, body mass index; BPA, bisphenol A; CpG, cytosine nucleotide followed by a guanine nucleotide; *EGFR*, epidermal growth factor receptor; EWAS, epigenome-wide association study; FDR, false discovery rate; GDM, gestational diabetes; *IGF2*, insulin-like growth factor 2; LC-MS/MS, liquid chromatography-mass spectrometry; *LINE-1*, long interspersed nuclear element 1; *LYRM2*, LYR motif containing 2; MeDIP, methylated DNA immunoprecipitation; *NR3C1*, nuclear receptor subfamily 2 group C member 1; *PDE4B*, phosphodiesterase 4B; PMD, partially methylated domain; POP, persistent organic pollutant; *PXT1*, peroxisomal testis enriched protein 1; *SAMe*, S-Adenosyl methionine; WGBS, whole-genome bisulfide sequencing.

**Table 2:** Placenta epigenetics, disease, and fetal outcome.

Study	Disease	Technique coverage	*n*	Placenta tissue	Finding
[109]	PE	Immunohistochemistry with antibodies to 5mC and 5hmC	10 PE, 10 control	Biopsy from central part of maternal side, trophoblast cell line	PE was associated with increased 5mC and decreased 5hmC in placenta. Hypoxic conditions resulted in increased 5mC and decreased 5hmC in trophoblasts.
[13]	PE	Infinium HM450K, EWAS	12 for VCTs, 8 for extravillous cytotrophoblasts	Cultured villous and extravillous cytotrophoblasts from first trimester placentas	The differentially methylated region in *DAXX* associated with PE is also associated with trophoblast differentiation. Methylation in this region was also positively associated with *DAXX* expression.
[110]	PE	Infinium HM450K, EWAS	19 PE, 17 control	Full-length biopsy	PE was associated with 989 differentially methylated probes, 80.7% of which were hypomethylated. 132 of associated probes were also associated with changes in gene expression, enriched for TGF-β pathway.
[111]	PE, early-onset or late-onset	Infinium HM450K, EWAS	13 early-onset PE, 16 late-onset PE, 20 preterm births, 27 fetal growth-restricted pregnancies, and 36 uncomplicated pregnancies	4 pooled biopsies from the fetal side of the placenta	Early-onset PE was associated with 869 differentially methylated probes when compared with preterm births, but there were no differences between early-onset PE group and restricted growth or uncomplicated groups. There were no differences between late-onset PE and any of the three non-PE groups.
[112]	PE, early-onset or late-onset	Infinium HM450K, EWAS	13 early-onset PE, 16 late-onset PE, 20 preterm births, 27 fetal growth-restricted pregnancies, and 36 uncomplicated pregnancies	4 pooled biopsies from the fetal side of the placenta	Early-onset PE was associated with hypomethylation of 6 CpG sites compared with the preterm birth group, differences in 2 CpGs compared with the restricted-growth group, and 6 CpGs compared with the uncomplicated group. There were no differences between late-onset PE and any of the three non-PE groups.
[113]	FGR	ChIP sequencing to measure H3K27ac occupancy	5 with FGR, 4 control. All participants had undergone a cesarean section.	4 pooled biopsies from random locations	FGR associated with 970 differential H3K27ac peaks (3.2% of total). As a group, genes near differential H3K27ac peaks showed differential expression.
[114]	IUGR and LGA	RRBS, EWAS	6 AGA 5 LGA 6 IUGR	Placenta tissue	More than 1,000 DMRs identified at FDR < 5%, DNA methylation change >15%. Two DMRs replicated in a previous Infinium HM27 dataset [115].
[116]	Preterm birth and LGA	HPLC, global DNA methylation	73 preterm, 73 term	Placenta tissue	Higher global DNA methylation levels (*p* < .05) in women delivering small for gestation age infants.
[117]	PE	Infinium HM450K, EWAS	48 PE pregnancies, separated into 4 subtypes based on transcriptome	Placenta tissue	DNA methylation explained some of the PE-subtype–specific expression patterns, but overall correlation between DNA methylation and gene expression was low.
[118]	Newborn neurobehavior	Infinium HM450K, EWAS	335 pregnancies	Term placenta tissue	Two CpG sites (near *FHIT* and *ANKRD11* genes) were associated with infant attention.
[119]	Neural tube defects	Infinium HM450K, EWAS	19 control, 22 spina bifida, and 15 anencephalic fetuses	Second trimester human placental chorionic villi	No association between placental DNA methylation and neural tube defects.
[120]	IUGR	Infinium HM450K, EWAS	8 severely growth-discordant monozygotic twin pairs	Term placenta tissue	IUGR associated DMRs at 8 gene promoters, including leptin receptor gene.
[121]	ASD at age 3	WGBS, global at single base resolution	24 ASD and 23 typically developing children	Term placenta tissue	A DMR at a putative fetal brain enhancer near *DLL1* was hypermethylation in the ASD group.
[64]	Extreme preterm	Infinium HM450K, EWAS	25 indicated preterm (placental dysfunction), 59 spontaneous preterm (infection, membrane rupture)	Placenta tissue at birth	250 differentially methylated probes at genes associated with neurodevelopment. 17 probes predicted cognitive function at age 10.
[122]	EOPE, LOPE, IUGR	Infinium HM450K, EWAS	Discovery cohort: 43 controls, 22 EOPE, 18 LOPE, 11 IUGR Validation cohort: 15 controls, 22 EOPE, 11 LOPE	Placenta tissue at birth	EOPE, but not LOPE and IUGR, was associated with widespread DNA methylation change (1,700 probes) compared with gestation-matched healthy pregnancies. Of these, 35% were also identified in the validation cohort.
[123]	Preterm, chorioamnionitis	Infinium EPIC, EWAS	44 preterm, 22 with chorioamnionitis	Chorionic villi, amnion, chorion at birth	Acute chorioamnionitis was associated with 66 differentially methylated probes. These were related to immune function.

Abbreviations: AGA, appropriate for gestational age; *ANKRD11*, ankyrin repeat domain 11; ASD, Autism Spectrum Disease; ChIP, chromatin immunoprecipitation; CpG, cytoside-guanine dinucleotide; *DAXX*, death domain associated protein; *DLL1*, delta-like canonical notch ligand 1; DMR, differentially methylated regions; EOPE, early-onset preeclampsia; EWAS, epigenome-wide association study; FDR, false discovery rate; FGR, fetal growth restriction; *FHIT*, fragile histidine triad diadenosine triphosphatase; HPLC, high performance liquid chromatography; H3K27ac, histone H3 lysine 27 acetylation; IUGR, intrauterine growth restriction; LGA, large for gestational age; LOPE, late onset preeclampsia; PE, preeclampsia; RRBS, reduced representation bisulfide sequencing; TGF-β, transforming growth factor β; VCT, villous cytotrophoblast; WGBS, whole genome bisulfide sequencing; 5hmC, 5-hydroxymethylcytosine; 5mC, 5-methylcytosine.

Over the past 5 years, there has been a shift from looking at maternal consumption (smoking, adiposity, and nutrition) towards exposures related to the natural and social environment, like plastics, chemicals, air pollution, other pollutants, and war trauma, reflecting the emerging broader scientific and societal interest in the impact of climate change and social issues on human health. We anticipate that as these climate change and societal issues continue to emerge, increased multidisciplinary research will be required to examine the impact of such exposures on many aspects of pregnancy, including placental development, epigenetic profile, and subsequent impact on placental and fetal health. We must also note that the majority of current studies are underpowered due to small sample size, and we urge that researchers consider EWAS power calculations and other considerations of study design prior to conducting such studies [55, 56]. It would be considerably beneficial to the field if the suppliers of the most commonly used platform made efforts to reduce the current excessive pricing of DNA methylation arrays relative to the current SNP-based genotyping equivalents.

### Exploiting the unique placental epigenome for noninvasive tracking of pregnancy health and outcome

Circulating fetal cell-free DNA in maternal blood is placental in origin, and has been used to noninvasively test for aneuploidy and disease-causing mutations [57, 58]. These tests depend on the detection of paternally inherited disease-causing SNPs that are not present in the mother. Early on, it was realised that placenta-specific DNA methylation patterns, such as the hypermethylation of the ras association domain-containing protein 1 (*RASSF1A*) promoter region [21], can be used as a positive control to rule out false negative results [59]. Since this pioneering work, Lo and colleagues used DNA methylation of fetal cell-free DNA for gestational dating, indicating that this technique has far-reaching potential [60]. However, replication of this finding is needed to confirm its usefulness in the diagnosis of gestational age. Recent studies have suggested that preeclampsia-specific DNA methylation patterns in the placenta, e.g., TIMP metallopeptidase inhibitor 3 (*TIMP3*) and *RASSF1A* promoters, can be detected in maternal circulation [61, 62]. However, no such test exists yet. It may also be possible to identify DNA methylation patterns associated with greater risk of preterm birth and small for gestational age (SGA) [63], or with their associated later morbidity [64, 65]. Such approaches have potential predictive utility through the analysis of circulating cell-free placenta-derived DNA, and are currently in development in several laboratories internationally [66, 67]. Key to this is identifying robust placenta methylation biomarkers associated with specific exposures and/or outcomes, which may not be feasible given the apparent low degree of methylation variation currently linked to most environmental exposures and outcomes (Table 1). It is now also possible to accurately reconstruct placental genome-wide DNA methylation by bisulfite sequencing of maternal plasma, which could allow the identification of epigenetic aberrations in the placenta without needing the placental sample itself [68].

### Single-cell approaches may overcome issues associated with placenta heterogeneity

Placenta is a heterogeneous tissue comprised of many cell types [69], some with varying chromosome copy numbers [70]. These characteristics make it difficult to interpret DNA methylation analysis, as well as limit biomarker use and understanding of molecular pathways and networks. Furthermore, the composition of these cell populations and corresponding DNA methylation profile may change throughout pregnancy [24, 71]. For the time being, it is likely that cohort-based EWAS will continue to use whole placental tissue because of the technical difficulties associated with isolating specific cell types on a large scale. Here, cell-specific DNA methylation profiles will aid research by providing signatures that can be used to deconvolute data and remove cell-specific signatures as variables in statistical analysis, similar to the way whole blood data are analysed [72]. However, deconvolution tools are currently limited to the number of different cell subtypes they include in their modelling, and further work is necessary to account for rare cell subtypes in analysis.

Recent advances in genome sequencing and cell sorting technology have enabled the unbiased analysis of all cell types in the human placenta. This is highlighted by the publication of several papers reporting single-cell RNA-seq profiles of the human fetal–maternal interface at the first and third trimesters in the last 3 years [69, 73–77]. These studies detected new subsets of cytotrophoblasts and EVTs, as well as cell subtypes previously not observed in placental villi [69, 73]. Continued application of single-cell RNA-seq to human placenta-derived cell populations, particularly over gestation, will greatly improve our understanding of how cell composition changes over time and how individual cells respond to changing environments, such as a shift in oxygen concentration. However, techniques for profiling epigenetic marks from low cell numbers are now available and in wide use [78, 79], including single-cell techniques for DNA accessibility [80], protein expression/phosphorylation [81], and combinations of marks [82–84]. Given the relative ease of obtaining placental tissue, these techniques should be readily applied to purified placental cells. Finally, placental organoid models that recapitulate villi structure and can produce ST and EVT cells have now been successfully established [85, 86], and unlike trophoblast cell lines [87], the organoids resemble first trimester trophoblasts in terms of their genome-wide DNA methylation patterns [86]. These novel models in combination with state-of-the-art molecular techniques represent a very powerful approach to provide hitherto unparalleled insights into human trophoblast differentiation and function.

## Conclusion

In this review, we highlighted the recent advances across the placental epigenetics field, which is now dominated by studies examining environmental influences. Given the close interaction between trophoblasts and immune cells, which make up to 40% of the feto-maternal interface, we anticipate the next few years will see epigenetic profiling of these resident immune cells. It will be interesting to see what PMDs look like in different placental cell types and how individual cells control the expression of hypomethylated retrotransposon promoters. Multi-omics approaches also have the potential to give us insight into the drivers and passengers involved in the interaction between the unique placental methylome and the environment.
